# Short time sports exercise boosts motor imagery patterns: implications of mental practice in rehabilitation programs

**DOI:** 10.3389/fnhum.2014.00469

**Published:** 2014-06-30

**Authors:** Selina C. Wriessnegger, David Steyrl, Karl Koschutnig, Gernot R. Müller-Putz

**Affiliations:** ^1^Laboratory of Brain-Computer Interfaces, Institute for Knowledge Discovery, Graz University of TechnologyGraz, Austria; ^2^BioTechMed-GrazGraz, Austria; ^3^Department of Psychology, University of GrazGraz, Austria

**Keywords:** motor imagery, functional magnetic resonance imaging, motor recovery

## Abstract

Motor imagery (MI) is a commonly used paradigm for the study of motor learning or cognitive aspects of action control. The rationale for using MI training to promote the relearning of motor function arises from research on the functional correlates that MI shares with the execution of physical movements. While most of the previous studies investigating MI were based on simple movements in the present study a more attractive mental practice was used to investigate cortical activation during MI. We measured cerebral responses with functional magnetic resonance imaging (fMRI) in twenty three healthy volunteers as they imagined playing soccer or tennis before and after a short physical sports exercise. Our results demonstrated that only 10 min of training are enough to boost MI patterns in motor related brain regions including premotor cortex and supplementary motor area (SMA) but also fronto-parietal and subcortical structures. This supports previous findings that MI has beneficial effects especially in combination with motor execution when used in motor rehabilitation or motor learning processes. We conclude that sports MI combined with an interactive game environment could be a promising additional tool in future rehabilitation programs aiming to improve upper or lower limb functions or support neuroplasticity.

## INTRODUCTION

Motor imagery (MI) and motor execution (ME) are promising strategies in motor skill learning and motor abilities rehabilitation. The term MI describes the mental simulation of voluntary movement without its actual execution (Jeannerod, 1995). It is widely recognized at the present time that the duration of MI usually correlates with the duration of real movements (temporal coupling) and that imagery of an action or its physical execution engages largely similar neural motor and motor related regions such as the supplementary motor area (SMA), the premotor cortex (PMC), the primary motor cortex (M1), posterior parietal regions (e.g., inferior and superior parietal lobe) the basal ganglia and the cerebellum (Lotze and Halsband, 2006; Guillot et al., 2008; Munzert et al., 2009). Several neuroimaging studies found that these areas are activated during both, MI and also ME (Jeannerod, 1994; Gerardin et al., 2000; Porro et al., 2000; Solodkin et al., 2004; Szameitat et al., 2007a; de Lange et al., 2008; Munzert et al., 2008; Munzert and Zentgraf, 2009; Zhang et al., 2011), thus both share the majority of networks especially the key area M1. However, Sharma and Baron (2013) recently reported a network, involving the ipsilateral PMC and the postcentral gyrus, which appears exclusive for MI. In recent years, MI has emerged as a promising, noninvasive technique to improve motor skill performance in rehabilitation programs, especially in stroke patients (Braun et al., 2006; Sharma et al., 2006; de Vries and Mulder, 2007; Ietswaart et al., 2011; Kaiser et al., 2012; Timmermans et al., 2013). As highlighted by several papers concerning the use of MI in rehabilitation (Jackson et al., 2001; Dijkerman et al., 2010; Malouin et al., 2013) there are marked differences in experimental designs and research protocols among the growing number of studies. Nevertheless the positive effects of MI on motor abilities rehabilitation are undisputable.

Furthermore MI is a widely used strategy for improving motor task performance and learning in a variety of sports (Brouziyne and Molinaro, 2005; Olsson et al., 2008). For example Olsson et al. (2008) investigated the role of task familiarity in relation to task complexity in a group of high jumpers and novices. They found that the activation of related motor regions strongly depends on a well-established motor representation from physical training. Moreover MI is a widely used paradigm to study cognitive aspects of action control and motor behavior in correlated brain structures (de Lange et al., 2008; Munzert et al., 2009). As demonstrated in animal models (e.g., Nudo et al., 1996) and in humans (Pascual-Leone et al., 1995; Lafleur et al., 2002; Jackson et al., 2003) the rehearsal of motor actions through physical and mental practice can induce brain changes (plasticity) associated with skill learning. For example Pascual-Leone et al. (1995), reported that the changes in cortical sensorimotor maps after mental training are similar to those obtained with physical training. Furthermore anatomical studies in nonhuman primates have shown that parts of the cingulate cortex directly project to M1 and the spinal cord, and it is thus likely that these premotor areas directly relate to the generation of movement (He et al., 1995). In past years, attention has been paid to how MI and physical training impacts motor function has attracted a lot of attention. Especially the effectiveness of MI training on behavior and neural mechanisms has been subject to recent functional magnetic resonance imaging (fMRI) studies (Jackson et al., 2003; Nyberg et al., 2006; Zhang et al., 2011). Zhang et al. (2011) showed that 2 weeks MI training could improve motor performance and induced brain functional alterations. This individual ability to imagine movements has already been studied by Guillot et al. (2008) in an fMRI study. They aimed to identify cortical patterns of MI in good and poor imagers who were selected by a broad testing procedure. Their results demonstrated that compared to skilled imagers, poor imagers also activate the cortico-cerebellar system during MI of sequential movements. Another study, by Munroe et al. (2000), showed that subject-dependent variables such as the ability to create vivid mental images also had effects on MI performance. This was also demonstrated by Lorey et al. (2011) in an fMRI study, who found increased perceived imagery vividness associated with increasing neural activity within the left putamen, the left PMC, the left parietal, primary and somatosensory motor cortex and the cerebellum. They found that increased vividness of movement imagery is strongly associated with neural activity in motor related areas. So the clarity and realism of the imagery experience could improve individual MI performance leading to stronger neural activation patterns. Many studies have been carried out focusing on the neural activation patterns during MI but with different paradigms. There are differences in the effectors that are used in the imagined action (e.g., hand, foot, mouth) or the complexity, ranging from simple finger tapping (Hanakawa et al., 2001, 2008; Nyberg et al., 2006; Zhang et al., 2011; Sharma and Baron, 2013) to walking (Bakker et al., 2007; Lee et al., 2011; Kalicinski and Raab, 2013) or sports (Owen et al., 2006; Olsson et al., 2008; Guillot et al., 2013). However, only a few studies have been done using more complex movements, the major part reporting MI patterns based on very simple movements. Our goal in the present study was thus to examine the impact of a very short term sports exercise on MI patterns. Athletes commonly use MI to refresh kinaesthetic memory between training sessions to maintain their performance level or for stabilizing complex routines (Rodgers et al., 1991; Murphy, 1994; Rushall and Lippman, 1998). They imagine their forthcoming performance in real time to “get a feeling” for how to respond to the requirements of a task (Munzert and Lorey, 2013). Another study performed by Szameitat et al. (2007a, b) investigated the functional neuroanatomical correlates of MI of complex everyday movements, e.g., eating or swimming. They found activation in the lateral and medial premotor cortices bilaterally, the left parietal cortex, primary sensorimotor cortices (SMC) and the right basal ganglia. The results demonstrated that MI of everyday movements drives a cortical network comparable to the one described for simpler movements, such as finger or hand tapping.

Based on the mentioned literature we hypothesized that after a short sports exercise, MI related brain activity increases due to the vividness of the previous executed motor exercise representation in memory. Furthermore we expected no influence of the effector, meaning that MI of soccer and MI of tennis will both elicit enhanced brain activity. We designed a more complex paradigm where participants played tennis or soccer in a virtual gaming environment between two MI tasks of the same sports. The two types of sports were chosen because they involve both effectors, are widely known, and reflect more complex movement procedures. To enable an easy integration of the motor exercise in daily life we have chosen a virtual gaming environment, namely Kinect. As a result the stronger activation of MI related areas after a short term sports intervention could enhance its application in future motor rehabilitation programs.

## MATERIALS AND METHODS

### PARTICIPANTS

Twenty three healthy right handed participants (15 male, 8 female, mean age 28.4 years, SD ± 4.3, range 19–39) took part in the experiment. Each participant was informed about the aim of the study and signed informed consent prior to the experiment. Additionally, each participant signed a further form after receiving information about risks and exclusion criteria of fMRI. The participants received compensation of €7.50 per hour and a CD of their personal anatomical brain scan. There were neither professional soccer nor tennis players amongst the participants. The experiment was conducted in compliance with the World Medical Association Declaration of Helsinki and the protocol was approved by the Ethics committee of the Medical University of Graz.

### DESIGN AND PROCEDURE

**FIGURE 1:**
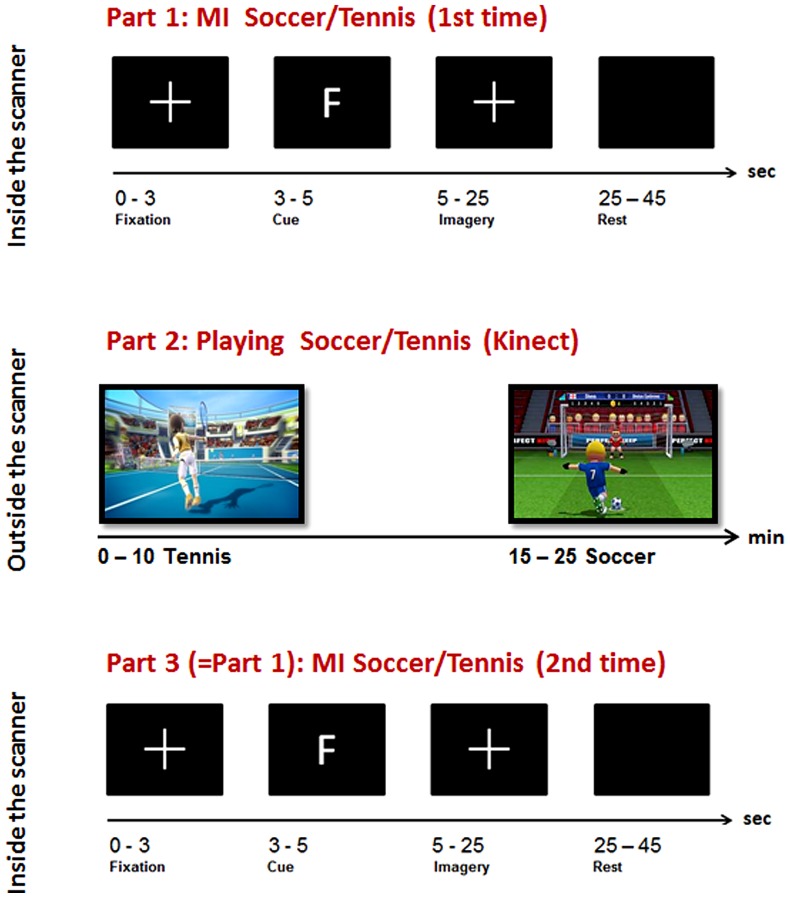
**Overview of the experimental paradigm: top: Part 1, Imagery task inside the scanner; middle: Part 2, sports exercise outside the scanner; bottom: Part 3, Imagery task inside the scanner**.

The experiment consisted of three parts (MI-ME-MI): while two parts of MI were performed inside the scanner, the execution condition was carried out outside the scanner (see **Figure 1**, part 1 and 3). For the MI task, two very common sports, tennis and soccer, were chosen because they furthermore integrate both effectors, namely hands and feet. To enable an easy integration of the motor exercise in daily life we have chosen a virtual gaming environment, namely Kinect.

During the general instruction on and information about the experiment participants were instructed to imagine playing soccer and tennis for 1 min with eyes open, each to become familiar with this kind of task. The imagery instruction was very concrete and similar for all participants. For soccer MI they had to imagine penalty kicks several times without running or interacting with other players. For tennis MI they had to imagine repeatedly returning balls. These MI instructions were chosen since they are compatible with the following sports exercise outside the scanner realized via Kinect. Before each imagery session inside the scanner an anatomical T1 scan was conducted lasting about 7 min. During this time period they watched a movie showing different landscapes. After that the first session inside the scanner started where they had to imagine playing soccer or tennis following a pseudo-randomized paradigm presented on a screen (**Figure 1**, part 1).

The type of imagery was indicated by an F (German = Fussball) for soccer and T for tennis. Each participant performed 14 (7 each condition) trials with 20 s MI of soccer or tennis and 20 s rest periods. The imagery periods were indicated by a white cross on the screen and the rest periods by a blank screen (see **Figure 1**, top).

**FIGURE 2:**
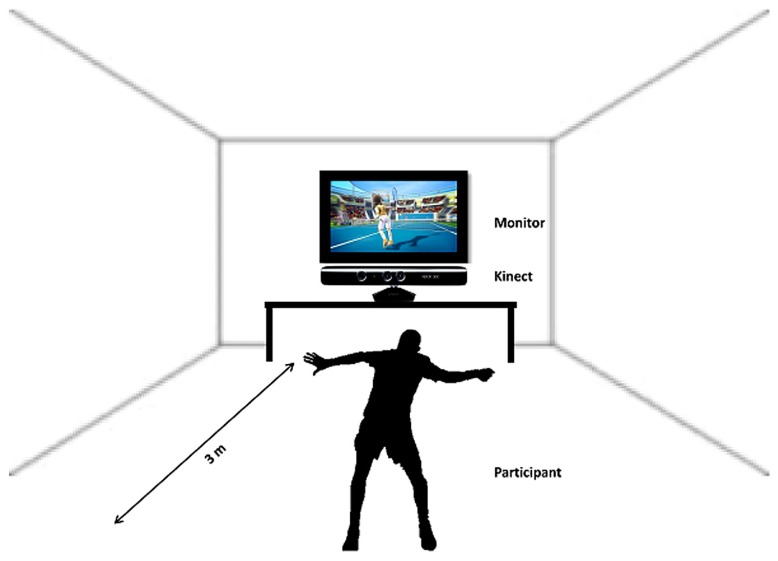
**Schematic illustration of the sports exercise, playing Tennis/Soccer via Kinect**.

After the participants had finished the first session of imagery inside the scanner the instructor guided them to the room where the next experimental part, the sports exercise, had to be performed. Following a short introduction the participants started to play soccer or tennis via “Kinect” (see **Figure 2**). This sports exercise was randomized among the subjects, some of them started playing soccer and others started with the tennis exercise.

Participants were instructed to imagine that they were the player (first person view) shown on the screen during the Kinect session. The total playing time lasted about 20 min (10 min soccer, 10 min tennis). Immediately after the execution task they again performed an imagery task inside the scanner. They were instructed to imagine playing soccer or tennis once more, just as in the session before the Kinect play (**Figure 1**, Part 3). An anatomical T1 scan was conducted prior to the experimental condition. Afterwards the same paradigm was started as in the first part of the experiment started. Again 14 trials of soccer and tennis imagery had to be performed in randomized order regarding the cues on the monitor. So, there was no difference between part 1 and part 3 (imagery inside the scanner). Since no MRI-compatible electromyographic (EMG) device was available during the measurements, an experimenter observed participants’ hands and feet via a scanner camera to ensure that they did not move during the MI conditions. The whole experiment lasted about 2 h including two scanning times of about 30 min.

### KINECT

Kinect is a motion sensing device by Microsoft for the Xbox 360 video game console and Windows PCs. It enables users to control and interact with the Xbox 360 without the need to touch a game controller through a natural user interface. The device features an “RGB camera, depth sensor and multi-array microphone running proprietary software,” which provide full-body 3D motion capture, facial recognition and voice recognition capabilities. The hands-free, full body control scheme of the Kinect makes it ideal for creating video games that get people active and moving. In addition, the Kinect’s camera can watch you move and record your movements, so it can give feedback on how much you’re moving or whether you’re doing a particular exercise correctly.

### MRI PROCEDURE

The fMRI recording was performed in a 3 T Siemens (Erlangen, Germany) Magnetom Skyra whole body scanner using a standard 32 channel head coil.

To minimize head movements, subjects’ heads were stabilized with foam cushions. Functional imaging was obtained using a BOLD sensitive (blood oxygenation level-dependent) T2^*^-weighted EPI-sequence (TR = 3000 ms, TE = 31 ms, flip angle = 90°, FOV = 240 mm). The acquisition consisted of 36 transverse slices with an isotropic spatial resolution of 3 mm providing coverage of the whole cerebral cortex. Additionally, structural T1-wighted images for each participant were collected using a MPRAGE sequence (176 sagittal slices; TR = 1560 ms, TE = 2.07 ms, with FOV 256 mm × 256 mm × 176 mm and a spatial resolution 1 mm × 1 mm × 1 mm, FOV = 256 mm). Stimuli were projected using an LCD projector (Nec) onto a projection wall positioned in front of the scanner, visible for the participants through a mirror mounted above the head coil. The paradigm was presented using a self-written script in MATLAB (The Mathworks Inc., Natick, MA, USA) and synchronized with MRI data acquisition.

### DATA ANALYSIS

Data were preprocessed and analyzed using SPM8 (Statistical Parametric Mapping; http://www.fil.ion.ucl.ac.uk/spm) implemented in MATLAB (The Mathworks Inc., Natick, MA, USA).

The first two functional images of each participant were discarded to allow for magnetic saturation. The remaining functional images were motion and slice acquisition time corrected, normalized to Montreal Neurological Institute (MNI) space and smoothed with a Gaussian kernel of 8 mm FWHM.

The statistical analysis was conducted on the basis of the general linear model as implemented in SPM. Model time courses for the two imagery conditions (Soccer and Tennis) as well as for the conditions “instruction” and the resting period were generated on the basis of the hemodynamic response function. Additionally six motion parameters were entered into the model as regressors of no interest.

To identify the location of brain areas involved in each task, one sample *t*-tests were used to contrast the imagery conditions before (PRE) and after (POST) the sports exercise (i) together (PRE: soccer versus tennis and POST: soccer versus tennis), (ii) separately (soccer POST versus soccer PRE and tennis POST versus tennis PRE) and (iii) overall (PRE: soccer and tennis versus POST: soccer and tennis). Results were obtained after initial thresholding at *P* < 0.001 uncorrected at the voxel level, followed by FWE (Family Wise Error) correction for multiple comparisons at the cluster level at *P* < 0.05. In the maps only clusters with spatial extent >30 were considered significant. A flexible factorial design analysis was performed to highlight differences in activation patterns between subjects and tasks. The flexible factorial analysis was chosen because multiple scans were performed for each subject. The analysis used a “subject by condition” design to model the interactions between subject and test condition (Imagery soccer or tennis) factors as well as to model global effects for each subject.

The activation maps were visualized using the xjView toolbox (http://www.alivelearn.net/xjview).

## RESULTS

**FIGURE 3:**
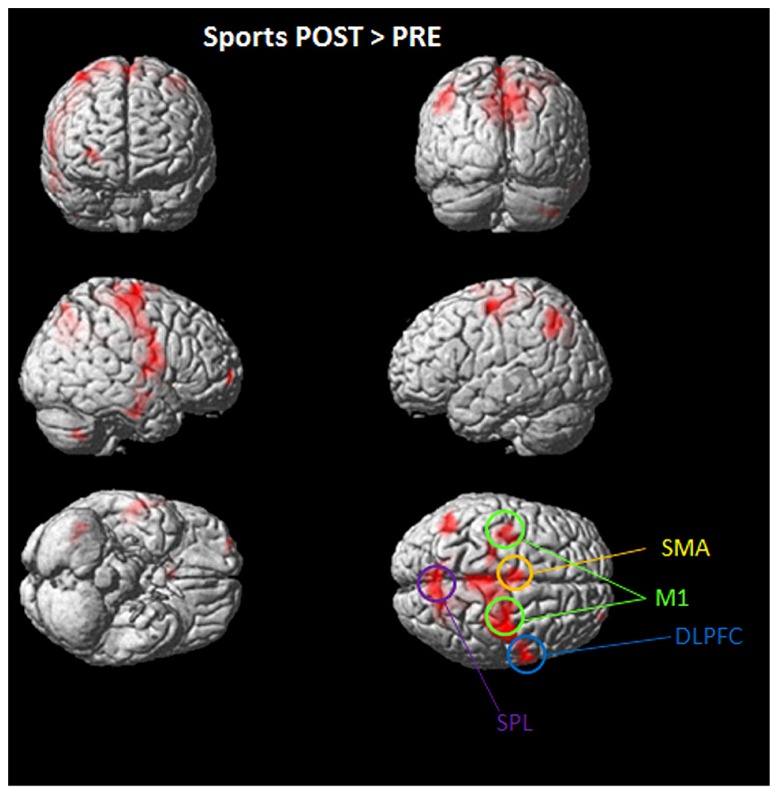
**Activated regions in direct comparisons “Sports POST > PRE” condition**. All regional activations above initial significance threshold *P* < 0.05 (FEW corrected) and extent (*k*_E_) of 30 voxels are depicted on a rendered MNI brain. DLPFC = dorsolateral prefrontal cortex; SPL = superior parietal lobule; M1 = primary motor cortex; SMA = supplementary motor area.

Overall significant activation increases were found in the POST condition (after playing Kinect) for soccer and tennis imagery. Utilizing subtraction analysis (the > indicates that comparisons showing higher values for the first condition are made), significant activation during the “Sports Imagery POST” minus the “Sports Imagery” PRE’ was observed in the right DLPFC, SMA, M1 bilateral, and SPL (**Figure 3**).

**FIGURE 4:**
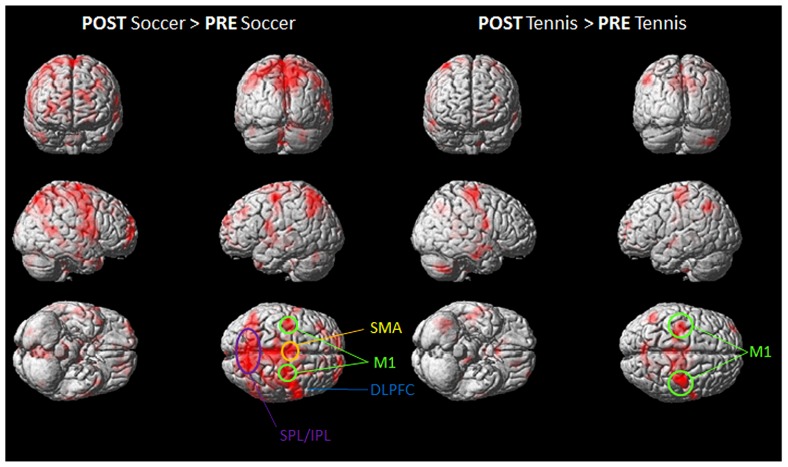
**Group activation maps showing activated brain regions in condition “POST Soccer > PRE Soccer (left side) and POST Tennis > PRE Tennis (right side).”** All regional activations above initial significance threshold *P* < 0.05 (FEW corrected) and extent (*k*_E_) of 30 voxels are depicted on a rendered MNI brain. DLPFC = dorsolateral prefrontal cortex; SPL = superior parietal lobule; IPL = inferior parietal lobule; M1 = primary motor cortex; SMA = supplementary motor area.

During soccer MI stronger activation was found in the SMA, the primary motor cortex (M1), DLPFC and the superior and inferior parietal lobe (see **Table 1** and **Figure 4**) after the physical exercise. By contrast, during tennis MI only parts of the posterior cingulate cortex and the primary motor cortex were significantly activated (**Figure 4**). However, subcortical regions like the cingulate cortex and precuneus also showed significant activation in both conditions (**Table 1**).

**Table 1:** Coordinates of peak activation during Imagery of Soccer and Tennis before (PRE) and after (POST) sports exercise.

Brain areas	Hem	*t* Value	*k*_E_	Coordinates of maximum *t* value		
				*x*	*y*	*z*
**PRE: soccer > tennis**					
Middle frontal gyrus	R	4.34	32	36	-46	4
**POST: soccer > tennis**						
Superior parietal lobe	L	4.57	178	-24	-55	61
**Soccer POST > PRE**						
Cingulum mid	L	6.06	164	-6	-22	31
Precuneus	R	5.03	359	12	-67	46
Precentral gyrus	R	5.3	230	42	-7	40
Paracentral lobule	L	4.84	95	0	-40	67
**Tennis POST > PRE**						
Cingulum Mid	R	5.05	239	9	-22	31
Precentralgyrus	R	4.24	62	39	-16	61
**Sports POST > PRE**						
Cingulum mid	L	7.24	78	-6	-25	31
Precuneus	R	6.53	54	15	-64	34

*MNI coordinates of peak activations for the comparisons between Soccer and Tennis POST and PRE condition; hemisphere (Hem), *t*-values, size of respective cluster (*k*_E_) at significance level *p*-value < 0.05. MNI coordinates denote the peak activation of voxels surviving correction for multiple comparisons for Soccer versus Tennis imagery in the POST and PRE condition (*P* < 0.05, FWE corrected; *k*_E_ > 30 voxels).*

**Figure 4** shows the mean activation contrasts of all participants for the comparison between soccer and tennis before (PRE) and after (POST) the execution task (playing soccer or tennis via Kinect).

Both MI tasks showed activation increases in M1, SMA, DLPFC with a greater degree for soccer MI. A further activation site was found for soccer exclusively within posterior parietal regions like the IPL and SPL.

**FIGURE 5:**
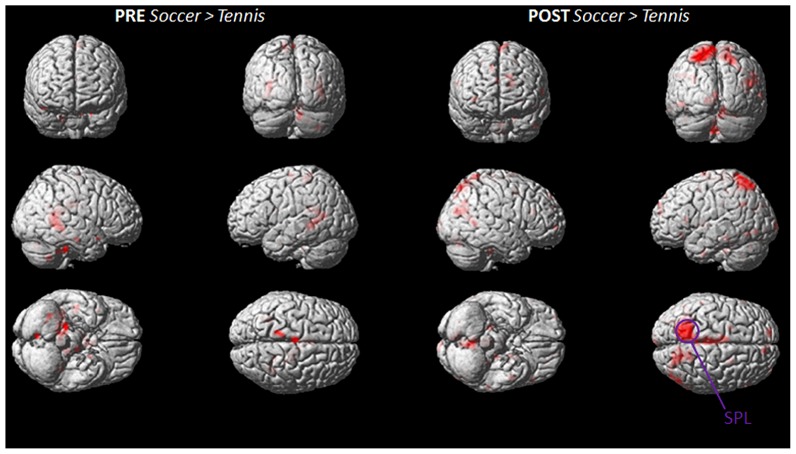
**Group activation maps showing activated brain regions in condition “PRE Soccer > Tennis (left side) and POST Soccer > Tennis (right side).”** All regional activations above initial significance threshold *P* < 0.05 (FEW corrected) and extent (*k*_E_) of 30 voxels are depicted on a rendered MNI brain. SPL = superior parietal lobule.

The stronger involvement of the SPL, during MI for soccer can be seen in **Figure 5**, which illustrates the imagery contrasts for soccer and tennis separately for the PRE and POST condition.

The inverse contrasts (soccer PRE > soccer POST/tennis PRE > tennis POST and PRE tennis > soccer/ POST tennis > soccer) showed no activated voxels above threshold.

## DISCUSSION

Our results showed that just 10 min of exercise influenced MI patterns, leading to enhanced activation in both MI tasks, with a stronger and more distributed network during the soccer task. To elucidate the difference in activation between the POST > PRE condition, we utilized subtraction analysis for soccer and tennis separately. This process highlighted the right DLPFC, the SMA and M1 for both sports but the SPL and IPL bilateral showed enhanced activation during imagery of soccer only. Guillot et al. (2008) have already provided evidence that motor related regions and the IPL and SPL were the common neural substrate of visual and kinaesthetic imagery. Although the general involvement of these areas has been further supported by other studies (Solodkin et al., 2004; Mizuguchi et al., 2013), the reason for a higher activation during soccer imagery in the present study remains unclear. One explanation might be that soccer is a more complex sport compared to tennis since it involves keeping one’s balance and working with one’s hands. For the current task, in particular, participants were instructed to execute and imagine penalty kicks the achievement of balance posing was a further challenge. Furthermore soccer seems to be a more motion-rich sport reflected in a more vivid memorized image which could also be reflected in the stronger activation pattern of the PMC and SMA while playing soccer. Since the SMA is known to be involved in planning or imagery of more complex movements (Porro et al., 1996; Nachev et al., 2008; Leek and Johnston, 2009) this further indicates that this type of exercise might have been more complex for the participants compared to the MI of Tennis.

It is not surprising that DLPFC is activated in both MI tasks, since it is known to be more activated during MI than actual execution (Vry et al., 2012). The observed data provided further evidence for the important role of the front-parietal networks in simulation of the actual execution during MI. (Mizuguchi et al., 2013).

M1 is often not activated during MI, even though MI and ME rely on similar structures and we were able to detect activation of the primary motor cortex (M1) during soccer and tennis. After examining 122 experiments (from 75 papers) Hétu et al. (2013) showed that only 22 (from 16 papers) reported primary motor cortex activation during MI (18%). As a result they found a large fronto-parietal network of consistent activations during MI spanning over both hemispheres. In the frontal lobes, regions consistently activated were bilateral inferior frontal gyri (IFG; including the pars opercularis), precentral gyrus (PcG), middle frontal gyrus (MfG), the SMA, and regions of the anterior insula. In the parietal lobes, the bilateral SPL and supramarginal gyrus (SMG) in addition to the IPL were consistently activated as in our study. Furthermore, consistently activated subcortical regions included the left putamen, right thalamus, and pallidum. Our results are in line with the literature (Zhang et al., 2011; Hétu et al., 2013) since the commonly reported motor related areas are involved during MI of soccer and tennis (see **Table 1**; **Figure 3**). The reason for the M1 activation in our study is that participants performed more complex movements which recruit many muscles resulting in a stronger activation of the motor homunculus compared to simple movements like finger tapping. Overall we found significantly stronger activation in motor related areas in the post condition indicating that the short time sports intervention enhances MI patterns. As reported by all participants it was definitely easier to imagine the requested type of sport after the exercise because of its vividness in memory. This finding could be important for future rehabilitation programs, e.g., in stroke therapy, using more attractive and vivid MI tasks instead of simple hand or foot movements which are less motivating and Also athletes do this type of MI every day to improve their motor skills, and the imagery of sports could be easily transferred and implemented in rehabilitation programs comprising recovery processes. Another important point is the use of MI training in daily practice since the basic ingredient of motor learning is a high number of repetitions (Pascual-Leone et al., 1995; Reiser et al., 2011). One possible way to increase mental repetitions in a stimulating fashion outside therapy sessions could be to use dynamic interactive applications such as the Kinect (Scherer et al., 2012; Lee, 2013). Comparable with the setup in the present study, a stroke patient, for example, could play tennis with his/her unaffected hand via an interactive game and afterwards imagine the same exercise in a relaxing situation also with his affected hand. Besides the attractive game situation he/she will also benefit from the positive effects of the combined practical and mental training session, as the findings of our study have shown.

Moreover the physical practice of actions seems to be a prerequisite for its imagery, meaning that if you cannot perform an action physically you will not be able to think it mentally. So when deciding to use imagery for rehabilitation it is important that you have already physically executed it since only then the functional equivalence can be expected and the imagery intervention will be successful (Olsson and Nyberg, 2010). Thus the proposed combination of using an interactive game and the sports imagery task could be beneficial for future rehabilitation programs.

Furthermore a close connection between imagery vividness and neural activation in motor related areas has been already shown by Lorey et al. (2011). They show that the clarity and realism of the respective imagery experience is crucial for increased neural activation. Our findings support their theory owing to the fact that imagining playing soccer or tennis after physical intervention is associated with a vivid maintenance of the image in working memory. Summarizing the suggested combination of sports exercise realized in a virtual environment with MI is promisingly due to its attractiveness, vividness in memory, high fun potential and usability.

Although the study delivered clear and focused results there are some limitations. Even though an experimenter observed the participants’ hands and feet during MI performance via a camera in the scanners room, in following studies additional EMG (electromyography) should be used as control of movement artifacts. Furthermore it would have been interesting to find out if the imagery of soccer is more vivid compared to tennis with the result of stronger neural activation. That is why the application of psychological evaluation tools, such as the Vividness of Movement Imagery Questionnaire (Roberts et al., 2008), would be valuable in follow up studies in order to assess subjective vividness of imagery. Moreover the investigation of an additional control session, participants only perform sports MI without ME, might underpin the present results.

In conclusion our findings are in accordance with previous studies suggesting that MI-based mental practice is effective because it activates a comparable cortical network as overt training. Furthermore we were able to demonstrate significant effects of short-term sports exercise on brain activity during MI of the same sports type with a higher activation pattern for soccer. This is an important result since it shows that just a few minutes of motor exercise on the eve of the imagery task leads to stronger cerebral activation in motor related areas. Although there is no proof of a long term effect, the mental practice presented combined with an interactive gaming environment could be a promising additional tool in future rehabilitation programs aiming to improve upper or lower limb functions or to support neuroplasticity.

## Conflict of Interest Statement

The authors declare that the research was conducted in the absence of any commercial or financial relationships that could be construed as a potential conflict of interest.
